# Lifestyle factors predict gout outcomes: Results from the NOR-Gout longitudinal 2-year treat-to-target study

**DOI:** 10.1136/rmdopen-2023-003600

**Published:** 2023-12-01

**Authors:** Till Uhlig, Lars Fridtjof Karoliussen, Joseph Sexton, Tore K Kvien, Espen A Haavardsholm, Hilde Berner Hammer

**Affiliations:** 1 Center for treatment of Rheumatic and Musculoskeletal Diseases (REMEDY), Diakonhjemmet Hospital, Oslo, Norway; 2 University of Oslo, Oslo, Norway

**Keywords:** gout, lipids, arthritis, therapeutics

## Abstract

**Objective:**

Gout is associated with lifestyle, body mass index (BMI) and comorbidities, including dyslipidaemia. We studied how in actively treated patients, anthropometric measures and lipid levels changed over 2 years and whether they predicted gout outcomes.

**Methods:**

Patients with a recent gout flare and elevated serum urate (sUA) received gout education and treat-to-target urate-lowering therapy over 1 year. Anthropometric measures with BMI, waist circumference (WC) and waist–height ratio (WHR) as well as lipid levels were measured yearly over 2 years. We examined whether baseline anthropometric measures and lipid levels were related to flares and to achieving the sUA target.

**Results:**

At baseline, patients (n=211) were with mean age of 56.4 years and 95% were male. Over 2 years, anthropometric measures were largely unchanged while cholesterol and low-density lipoprotein cholesterol (LDL-C) were reduced at year 1. Anthropometric measures were associated with presence of tophi. Higher baseline WC (OR: 0.96 per cm, 95% CI: 0.93 to 0.99) decreased and high level of high-density lipoprotein cholesterol (OR: 5.1 per mmol/L, 95% CI: 1.2 to 22.1) increased the chance of sUA target achievement at year 2. High LDL-C (OR: 1.8 per mmol/L, 95% CI: 1.2 to 2.6) predicted the chance of having a gout flare during year 2.

**Conclusion:**

In actively treated patients with gout, anthropometric measures were largely unchanged over 2 years and lipid levels were reduced. High WC and lipid levels predicted unfavourable gout outcomes after 2 years.

**WHAT IS ALREADY KNOWN ON THIS TOPIC**

- Lifestyle factors are related to the incidence and burden of gout.

**WHAT THIS STUDY ADDS**

- After an educational consultation and urate-lowering therapy of gout the body mass index was largely unchanged over 2 years, while lipid levels decreased. Waist circumference and high lipid levels contribute to poor disease outcomes such as flares and not achieving the treatment target.

**HOW THIS STUDY MIGHT AFFECT RESEARCH, PRACTICE OR POLICY**

- Lifestyle, anthropometric measures and lipids are modifiable factors related to disease outcomes in gout with tophi, long-term flares, and low serum urate.

Gout is a frequent inflammatory joint disease with rising prevalence^1 2^ and a considerable disease burden worldwide.^3 4^ The disease manifests with high serum urate (sUA) as well as painful episodical flares^5^ and has impact on health-related quality of life.^6–8^

Obesity and weight gain are strong risk factors of gout in men.^9 10^ Body mass index (BMI) is a well described risk factor of gout,^11^ and other anthropometric measures such as waist circumference (WC) and waist/height ratio (WHR)^12^ are also associated with gout. Among anthropometric measures BMI may have the best predictive ability for gout risk in men and WC in women.^13^

Overweight and obesity are frequent in gout; obesity and hyperlipidaemia are also part of the metabolic syndrome. In the general population, diet explains very little of the variation in sUA levels compared with genetic factors and the interaction between genes and diet.^14 15^ However, a lifestyle with sugar-sweetened beverages may lead to elevated sUA in those with high BMI.^16 17^

Treatment recommendations for gout include non-pharmacological and urate-lowering therapy (ULT) to reduce sUA and prevent flares as well as other consequences of gout.^18–20^ Recommendations on lifestyle also aim to lower sUA. Cross-sectionally, high BMI is associated to not achieving the sUA treatment target,^21^ as is high alcohol consumption, hyperlipidaemia and increased WC.^22^ Further, some evidence supports weight loss for improved gout outcomes.^23^ Very little research from longitudinal studies examines whether recommendations on life-style changes and ULT lead to improved gout outcomes, that is, absence of flares and successful achievement of the sUA target.

We thus set out to study lifestyle in patients with gout, and how anthropometric measures and lipid levels changed over 2 years in patients who had received education with a study nurse and intensive ULT. We further examined whether these modifiable lifestyle factors could predict disease severity outcomes with tophus, as well as flares during year 2 and achievement of the sUA target at 2 years.

## Methods

### Study design and participants

**Figure 1:**
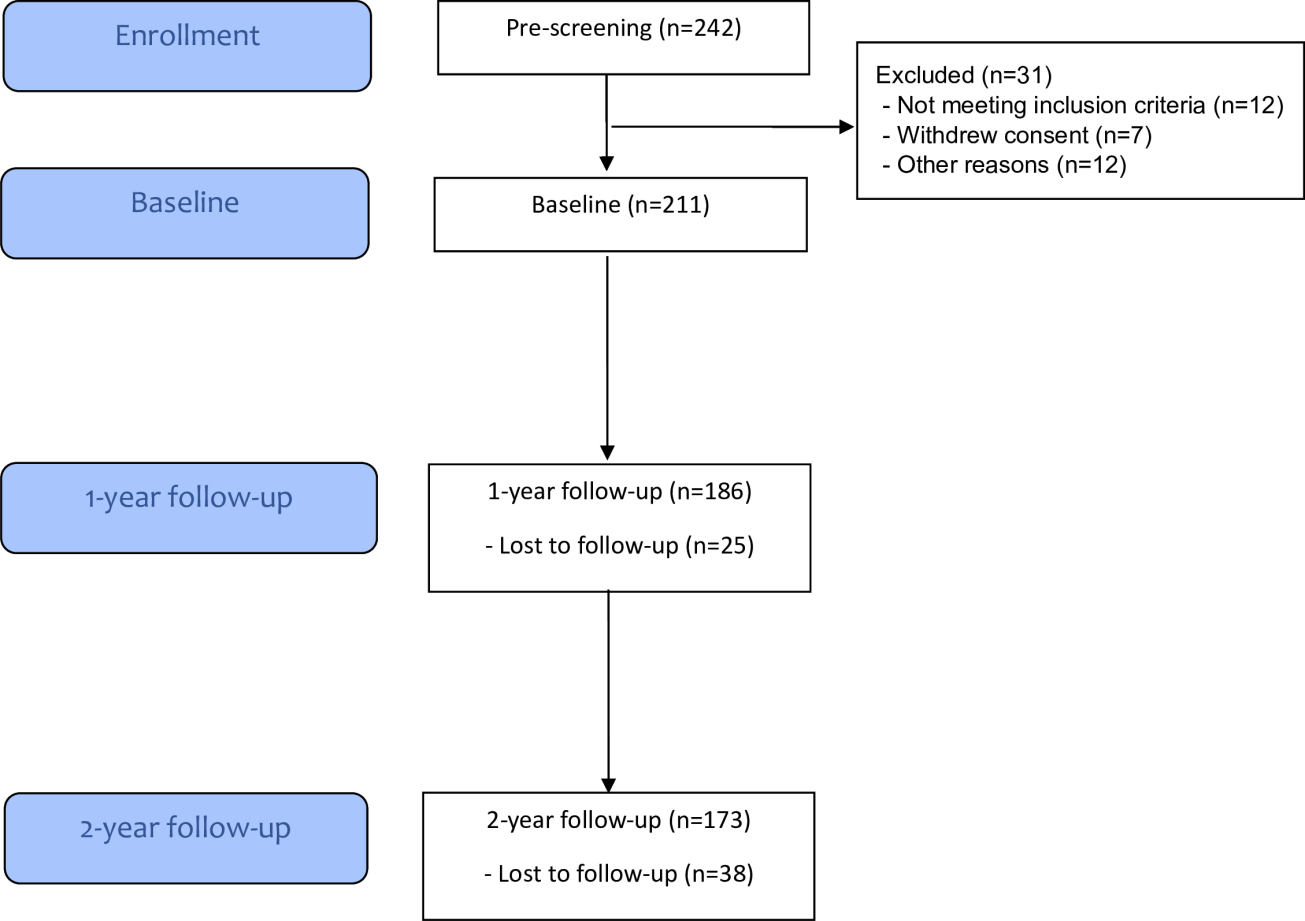
Flow diagram of NOR-Gout participants.

NOR-Gout (Gout in Norway) study is a prospective observational study performed in a hospital-based rheumatology clinic.^24 25^ All included patients had crystal proven gout and fulfilled the ACR/EULAR classification criteria for gout.^26^ Participants were identified during an acute clinical gout flare after examination in the rheumatology outpatient clinic. Patients were prescreened, contacted by a study nurse and scheduled a few weeks later for a comprehensive baseline rheumatology examination. They were required to have increased sUA (>360 µmol/L) at inclusion and started ULT with allopurinol or febuxostat, if intolerant to allopurinol, with frequent follow-up visits during the first year and a final visit after year 2 (figure 1). During this treat-to-target strategy, ULT was escalated to achieve sUA <360 µmol/L (or <300 µmol/L if clinical tophi were present) as recommended in international recommendations.^19 27^ All patients started simultaneously with colchicine for 3–6 months. Patients had at baseline one individual consultation with a trained rheumatology research nurse. This consultation comprised the following concepts which previously had been found helpful in the clinic:

- Gout disease and disease process, diagnosis, causes, recommended treatments and disease control.
- Lifestyle advocation on exercise, weight reduction and alcohol consumption.
- Patient expectations, advice when to get additional help and advice on coping

A brochure addressing diet was also provided. During follow-up consultations lifestyle aspects could be further informally addressed by nurse or patient on a visit-to-visit basis.

The study (ACTRN12618001372279) was registered at https://www.anzctr.org.au/Trial/Registration/TrialReview.aspx?id=374171. It was approved by the Regional Ethics Committee for Medical and Health Research Ethics Eastern Norway (reference number 2015/990), included patient representatives in project planning and was performed in accordance with the Declaration of Helsinki and Good Clinical Practice guidelines. All patients provided written informed consent. The sponsor of the study was Diakonhjemmet Hospital.

### Data collection

All patients were assessed by a study nurse and a rheumatologist at baseline, after 3 and 6 months, 1 and 2 years and monthly by the study nurse until the sUA target was achieved. At baseline, patients reported age, gender, ethnicity, marital status, highest level of education, family history for gout, disease duration, and lifestyle.

#### Lifestyle, anthropometric measures and laboratory values

Lifestyle for smoking, alcohol consumption, sugar-sweetened drinks and physical activity were patient-reported at baseline. Anthropometric factors were measured at baseline, year 1 and year 2 with BMI (kg/m^2^), WC in centimetres and WHR. WHR was defined as the ratio of waist to height, WC was measured at the mid-level between the iliac crest and the last rib.

Smoking was categorised as daily smoker or not, alcohol use at least weekly versus less, sugar-sweetened soft drink consumption as at least one glass daily versus not. Physical activity was categorised as present if patients stated physical activity over at least 30 min minimum three times per week.

BMI categories were normal weight (<25 kg/m^2^), overweight (≥25 kg/m^2^ to <30 kg/m^2^) and obesity (≥30 kg/m^2^). WC in men below 94 cm was defined as 'low risk', 94–102 cm as 'high risk' and more than 102 cm as 'very high risk'; for women, below 80 cm the risk was considered low, 80–88 cm high and more than 88 cm very high.^13^

Laboratory lipid values included total cholesterol, triglycerides, high-density lipoprotein cholesterol (HDL-C) and low-density lipoprotein cholesterol (LDL-C), all in mmol/L at baseline, year 1 and year 2. Kidney function was during all clinical visits assessed with creatinine (µmol/L) and estimated glomerular filtration rate (eGFR) in mL/min per 1.73 m^2^; erythrocyte sedimentation rate (ESR) in mm/hour, and sUA in µmol/L were examined.

#### Clinical outcomes

Gout outcomes in this study were at baseline tophaceous disease, achievement of the sUA target (<360 µmol/L) at year 2 and occurrence of a flare during year 2.

sUA was measured at all clinical visits during the study. At every clinical visit the patient self-reported gout flares since the last visit during a structured interview with a trained study nurse. If in doubt, patient and study nurse discussed whether an experienced episode with pain or swelling was to be defined as a gout flare or not. Information on number of flares ‘ever’ and ‘during the last year’ (before the recent study, ie, entry flare) was collected as well as pain severity during the most recent and the strongest flare (0–10 numerical rating scales), with 0=no pain and 10=unbearable pain. Occurrence of flares was also recorded between year 1 and 2.

For comorbidities the Self-Administered Comorbidity Questionnaire (SCQ) was used (score range: 0–36)^28^; it includes 12 medical problems, allocating 1 point in each problem for presence, receiving treatment, and causing a functional limitation.

Clinical assessments included 44 swollen and tender joint counts and presence of subcutaneous tophi was assessed.

### Statistical analysis

We applied descriptive statistics for baseline variables. For longitudinal analyses of anthropometric and laboratory analyses over 2 years, we applied paired t-test and χ^2^ test between time points. Groups with and without disease severity outcomes were compared with independent samples t-test and χ^2^ test. Categories for BMI and the WC across 2 years were compared with Friedman’s analysis of variance.

Demographic and disease-related variables (age, gender, disease duration and the comorbidity score) and lifestyle, including anthropometric measures and laboratory values were analysed as independent variables, whereas gout outcomes (flare during year 2 and sUA target achievement at year 2) were dependent variables. In multivariable logistic regression analyses these candidate variables were adjusted for age, gender, disease duration and comorbidity score. Variables with p<0.15 were then included in further multivariable model building where final models retained statistically significant variables (p<0.05), adjusted for age, gender, disease duration and comorbidity score. Dependent variables in two final multivariable logistic regression models were flare occurrence during year 2, and achievement of the sUA target <360 µmol/L at year 2. If several independent anthropometric or lipid variables contributed statistically significantly to the final models, we retained the variable contributing to best goodness to fit in the model according to the log-likelihood value.

No adjustments were made for missing data or multiple comparisons in the descriptive analyses. Analyses were performed with IBM SPSS statistics (V.29; IBM Corp, Armonk, New York, USA).

## Results

### Study population

Patients were predominantly male subjects, with mean age 56.4 years (SD: 13.7 years) and mean disease close to 8 years (table 1). Subcutaneous tophi were at baseline present in 16.6% of patients. At the end of the treat-to-target period at year 1, 85.5% of patients were at the predefined treatment target (sUA <360 µmol/L) and 78.6% at year 2. sUA decreased from mean 500 µmol/L at baseline to 311 µmol at 1 year and 324 µmol/L at year 2. During the second year 26.0% of patients had a gout flare.

**Table 1:** Patient characteristics

	N	Value
Age, mean±years	211	56.4 (13.7)
Male	201/211	95.3%
Caucasian	183/202	90.6%
Disease duration, mean±years	204	7.8 (7.6)
College education	118/206	57.3%
Working at baseline	134/208	64.4%
Comorbidity score, mean±SD	210	3.7 (3.2)
Smoking daily	23/208	11.1%
Alcohol use at least weekly	128/207	61.8%
Sugar-sweetened drink daily	80/207	38.6%
Physical activity at least three times weekly	63/207	30.4%
Body mass index, mean±SD kg/m^2^	211	28.8 (4.5)
Subcutaneous tophus	35/211	16.6%
Allopurinol use ever at baseline	31/211	14.7%
Allopurinol use month 12	163/186	87.6%
Febuxostat use month 12	23/186	12.4%)
Serum urate baseline, ±SD µmol/L	211	500 (77)
Previous flare last 12 months	151/206	73.4%
Strongest joint pain ever, mean±SD	208	8.4 (1.6)
Joint pain last flare, ±SD	207	7.5 (5.5)

### Anthropometric measures and laboratory values over 2 years

**Figure 2:**
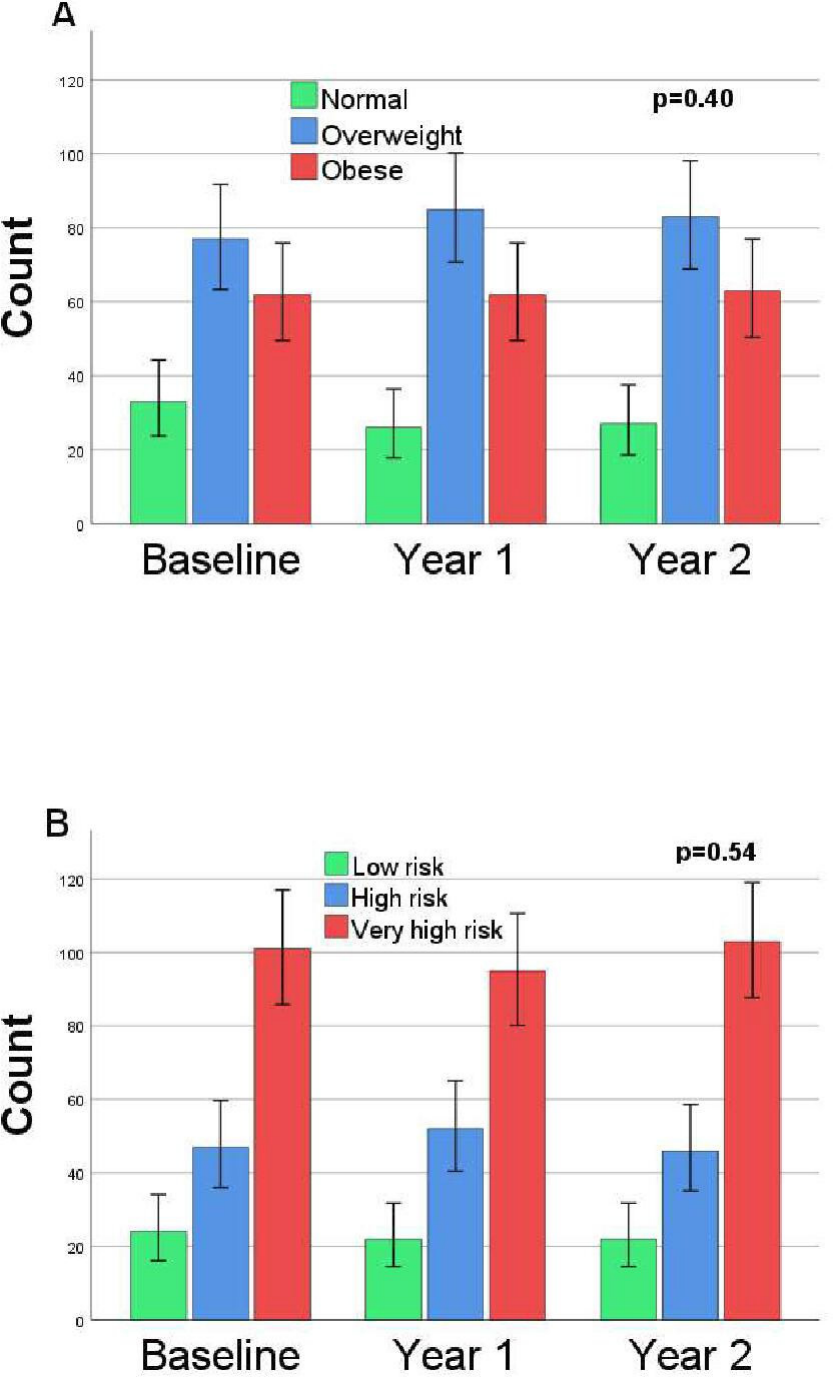
Categories for (A) body mass index and (B) waist–height ratio over 2 years with counts and 95% CIs.

There were small changes for anthropometrics measures, BMI was mainly unchanged over 2 years, but with a slight, statistically significant increase, from 28.78 to 29.16, p=0.018 (table 2), and no statistically significant changes were seen for WC and WHR. Percentages for BMI categories did not change over 2 years and are presented in figure 2. Lipid levels showed a decrease for cholesterol and LDL-C during year 1, and levels were then maintained during year 2. Kidney function measured with creatinine and eGFR showed small changes, and ESR was reduced (table 2).

**Table 2:** Anthropometric measures and laboratory values over 2 years

	Baseline (n=211)	Year 1 (n=186)	Year 2 (n=173)	P value Year 1 vs baseline	P value Year 2 vs baseline	P value Year 2 vs year 1
Anthropometric measures						
Body mass index (kg/m^2^)	28.78 (4.55)	29.06 (4.57)	29.16 (4.62)	0.09	**0.018**	0.08
Waist circumference (cm)	105.43 (12.49)	105.50 (12.62)	105.91 (12.31)	0.13	0.30	0.07
Waist–height ratio	0.586 (0.070)	0.585 (0.070)	0.588 (0.069)	0.11	0.32	0.07
Laboratory values						
Cholesterol (mmol/L)	5.23 (1.33)	4.41 (1.18)	4.38 (1.18)	**<0.001**	**<0.001**	0.49
HDL-C (mmol/L)	1.27 (0.38)	1.24 (0.36)	1.24 (0.37)	0.26	0.26	0.16
LDL-C (mmol/L)	2.94 (1.07)	2.34 (0.99)	2.54 (0.95)	**<0.001**	**<0.001**	**<0.001**
Triglycerides (mmol/L)	2.26 (1.36)	2.12 (1.26)	2.20 (2.00)	0.11	0.34	0.33
Creatinine (µmol/L)	96.3 (18.4)	94.7 (19.4)	93.9 (21.1)	0.07	**0.023**	0.16
eGFR (mL/min per 1.73 m^2^)	78.0 (18.8)	78.8 (19.1)	79.0 (19.8)	0.09	**0.040**	0.21
Erythrocyte sedimentation rate (mm/hour)	14.5 (14.2)	10.4 (10.5)	10.7 (10.4)	**<0.001**	**<0.001**	0.31
Serum urate (µmol/L)	500 (77)	311 (48)	324 (70)	**<0.001**	**<0.001**	**0.015**

Values are mean (±SD). P value with paired samples t test. Statistically significant values are in bold.

eGFR, electronic glomerular filtration rate; HDL-C, high density lipoprotein cholesterol; LDL-C, low density lipoprotein cholesterol.

### Lifestyle, anthropometric measures and laboratory values in bivariate analyses with tophus and flare

Patients with tophus had higher age, disease duration, creatinine and lower eGFR than patients without tophus at baseline. Tophi were associated with high WC and WHR (table 3). Higher levels for cholesterol and LDL-C were seen in patients with a flare versus those without a flare during year 2.

**Table 3:** Lifestyle, anthropometrical measures and laboratory values at baseline associated with clinical tophus at baseline and flares during the study

	Tophus at baseline			Serum urate target achieved (<360 µmol/L) year 2			Flare during year 2		
	Tophus not present (n=176)	Tophus present (n=35)	P value	Yes (n=136)	No (n=37)	P value	No flare (n=128)	Flare (n=45)	P value
Age, mean±years	55.3 (13.7)	61.9 (13.5)	**0.004**	58.3 (13.1)	51.6 (14.1)	**0.004**	56.8 (13.9)	57.1 (13.0)	0.45
Disease duration, mean±years	7.1 (6.7)	11.7 (10.5)	**<0.001**	8.3 (8.9)	8.0 (7.8)	0.40	8.1 (8.2)	8.8 (6.5)	0.31
Comorbidities, mean±SD	3.7 (3.3)	3.8 (2.9)	0.39	3.9 (3.2)	3.2 (3.7)	0.15	3.7 (3.3)	3.8 (3.4)	0.45
Lifestyle									
Smoking daily	21 (12.1)	2 (5.7)	0.59	10 (7.5)	5 (13.9)	0.58	14 (10.9)	1 (2.4)	0.12
Alcohol ≥weekly	106 (61.6)	22 (62.9)	0.89	85 (63.9)	19 (52.8)	0.22	80 (63.0)	24 (57.1)	0.50
Sugar-sweetened soft drink daily	70 (40.5)	10 (29.4)	0.23	44 (33.1)	21 (58.3)	**0.006**	49 (38.6)	16 (38.1)	0.96
Physical activity >3 times weekly	53 (30.8)	10 (28.6)	0.79	45 (33.1)	10 (29.4)	0.68	37 (29.1)	18 (41.9)	0.12
Anthropometric measures									
Body mass index, mean±kg/m^2^	28.6 (4.5)	29.8 (4.7)	0.08	28.4 (4.3)	30.7 (5.9)	**0.003**	29.2 (4.5)	28.1 (4.7)	0.10
Waist circumference, mean±cm	104.5 (12.4)	110.2 (12.1)	**0.007**	104.5 (12.0)	110.0 (11.6)	**0.007**	106.0 (12.0)	104.5 (12.7)	0.23
Waist-to-hight ratio, mean±SD	0.58 (0.07)	0.61 (0.07)	**0.014**	0.58 (0.07)	0.61 (0.07)	**0.01**	0.59 (0.07)	0.58 (0.07)	0.24
BMI category (kg/m^2^)			0.30			**0.03**			0.15
Normal <25	38 (21.7)	6 (17.1)		29 (21.5)	4 (10.8)		20 (15.7)	13 (28.9)	
Overweight 25–30	81 (46.3)	13 (37.1)		64 (47.4)	13 (35.1)		60 (47.2)	17 (37.8)	
Obese >30	56 (32.0)	16 (45.7)		37 (32.2)	28 (40.0)		77 (37.0)	15 (33.3)	
Waist circumference category			**0.03**			0.07			0.38
Normal	27 (15.4)	2 (5.7)		23 (17.0)	1 (2.7)		15 (11.8)	9 (20.0)	
Increased	55 (31.4)	6 (17.1)		37 (27.4)	10 (27.0)		35 (27.6)	12 (26.7)	
High	93 (53.1)	27 (77.1)		75 (55.6)	26 (70.3)		77 (60.8)	24 (53.3)	
Laboratory values									
Cholesterol, mean±mmol/L	5.2 (1.3)	5.3 (1.6)	0.43	5.2 (1.3)	5.1 (1.1)	0.36	5.0 (1.2)	5.6 (1.2)	**0.008**
HDL-C, mean±mmol/L	1.27 (0.39)	1.26 (0.29)	0.46	1.30 (0.41)	1.13 (0.28)	**0.01**	1.27 (0.40)	1.25 (0.36)	0.37
LDL-C, mean±mmol/L	2.95 (1.09)	2.89 (0.98)	0.39	2.94 (1.11)	2.87 (1.01)	0.38	2.76 (1.04)	3.39 (1.09)	**<0.001**
Triglycerides, mean mmol/L	2.26 (1.29)	2.28 (1.66)	0.46	2.18 (1.24)	2.56 (1.46)	0.057	2.24 (1.30)	2.29 (1.30)	0.43
Creatinine, mean µmol/L	94 (18)	105 (21)	**<0.001**	98 (18)	91 (15)	**0.018**	95 (17)	99 (20)	0.07
eGFR (mL/min per 1.73 m^2^	80 (90)	68 (17)	**<0.001**	76 (18)	85 (11)	**0.002**	78 (18)	75 (18)	0.16
ESR, mean±mm/hour	14.5 (14)	14.7 (15)	0.47	14 (14)	16 (15)	14 (14)	14 (14)	17 (15)	0.11
Serum urate, mean±µmol/L	489 (77)	506 (81)	0.30	492 (77)	521 (93)	492 (77)	496 (81)	504 (78)	0.28

Values are numbers (%) unless indicated otherwise. t-test or Pearson’s χ^2^ or Fisher’s exact test. Statistically significant p values are in bold.

eGFR, electronic glomerular filtration rate; ESR, erythrocyte sedimentation rate; HDL-C, high density lipoprotein cholesterol; LDL-C, low density lipoprotein cholesterol.

### Lifestyle, anthropometric measures and laboratory values in bivariate analyses with sUA target

The only bivariate association between lifestyle and achievement of the target sUA was more frequent intake of sugar-sweetened soft drink daily in patients not achieving the sUA target at year 2 (58.3% vs 33.1%, p=0.006, table 3). Baseline anthropometric measures such as BMI, WC and WHR were higher in patients not achieving the sUA outcome at year 2 (table 3). Baseline kidney function was lower creatinine and higher eGFR levels in patients at target year 2compared to those patients who were not.

### Independent prediction of 2-year sUA target and flare

Using multivariable analyses several variables at baseline were related with the 2-year sUA target and flare outcomes when adjusting for age, gender, disease duration and comorbidities. In adjusted logistic regression analyses daily intake of sugar-sweetened soft drinks, as well as all anthropometric measures (higher BMI, WC and WHR) reduced the OR of achieving the 2-year sUA target, while higher HDL-C increased the OR (table 4). In the final, fully adjusted logistic regression model only low WC, high HDL-C and low eGFR increased the OR of reaching the target sUA at year 2, and the statistical relationship with sugar-sweetened soft drinks was lost.

**Table 4:** Baseline risk of achieving 2-year serum urate target using logistic regression analyses

	Adjusted* OR (95% CI)	P value	Final model OR (95% CI)	P value
Lifestyle				
Smoking daily	0.42 (0.11 to 1.54)	0.19		
Alcohol weekly vs less	1.34 (0.59 to 3.02)	0.48		
Sugar-sweetened soft drink daily	0.40 (0.18 to 0.89	**0.024**		
Physical activity >3 times weekly	1.27 (0.55 to 1.96)	0.58		
Anthropometrical measures				
Body mass index (kg/m^2^)	0.91 (0.84 to 0.99)	**0.023**		
Waist circumference (cm)	0.96 (0.93 to 0.99)	**0.008**	0.96 (0.93 to 0.99)	**0.018**
Waist–height ratio	0.001 (0 to 0.22)	**0.014**		
Laboratory values				
Cholesterol (mmol/L)	1.14 (0.82 to 1.60)	0.43		
HDL-C (mmol/L)	4.32 (1.13 to 16.46)	**0.032**	5.08 (1.16 to 22.14)	**0.031**
LDL-C (mmol/L)	1.09 (0.73 to 1.61)	0.68		
Triglycerides (mmol/L)	0.87 (0.66 to 1.15)	0.32		
Creatinine (µmol/L)	1.02 (0.99 to 1.05)	0.13		
eGFR (mL/min per 1.73 m^2^)	0.98 (0.95 to 1.00)	0.11	0.97 (0.94 to 1.00)	**0.042**
ESR (mm/hour)	0.95 (0.95 to 1.00)	0.18		
Serum urate (µmol/L)	1.00 (0.99 to 1.00)	0.13		

*Adjusted for age, gender, disease duration and comorbidities. Statistically significant p values are in bold.

eGFR, estimated glomerular filtration rate; ESR, erythrocyte sedimentation rate; HDL-C, high density lipoprotein cholesterol; LDL-C, low density lipoprotein cholesterol.

Flares observed during year 2 were in the final regression model independently only predicted by high LDL-C levels (table 5). Cholesterol was also an independent predictor if entered instead of LDL-C.

**Table 5:** Baseline risk of a flare during year 2 using logistic regression analyses

	Adjusted* OR (95% CI)	P value	Full model* OR (95% CI)	P value
Lifestyle				
Smoking daily	0.19 (0.02 to 1.49)	0.11		
Alcohol weekly vs less	0.80 (0.38 to 1.72)	0.57		
Sugar-sweetened soft drink daily	0.97 (0.46 to 2.06)	0.93		
Physical activity >3 times weekly	1.80 (0.86 to 3.77)	0.12		
Anthropometrical measures				
Body mass index (kg/m^2^)	0.95 (0.87 to 1.03)	0.24		
Waist circumference (cm)	0.99 (0.96 to 1.02)	0.50		
Waist–height ratio	0.15 (0.001 to 37.9)	0.50		
Laboratory values				
Cholesterol (mmol/L)	1.46 (1.08 to 1.98)	**0.014**		
HDL-C (mmol/L)	0.84 (0.31 to 2.03)	0.74		
LDL-C (mmol/L)	1.79 (1.22 to 2.62)	**0.003**	1.79 (1.22 to 2.62)	**0.003**
Triglycerides (mmol/L)	1.06 (0.81 to 1.40)	0.66		
Creatinine (µmol/L)	1.01 (0.99 to 1.03)	0.34		
eGFR (mL/min. per 1.73 m^2^)	0.99 (0.96 to 1.01)	0.31		
ESR (mm/hour)	1.01 (0.99 to 1.04)	0.37		
Serum urate (µmol/L)	1.00 (1.00 to 1.1)	0.59		

*Adjusted for age, gender, disease duration and comorbidities. Statistically significant p values are in bold.

ESR, erythrocyte sedimentation rate; HDL-C, high density lipoprotein cholesterol; LDL-C, low density lipoprotein cholesterol.

In sensitivity analyses, we applied unadjusted logistic regression analyses to study if the intensity of ULT at the end of year 1 was related to sUA target achievement at year 1 and also to flare occurrence at year 2. Compared with a reference allopurinol dose of 100 mg at month 12, higher allopurinol dose (OR: 3.10, 95% CI: 0.86 to 11.20) or febuxostat (OR: 5.25, 95% CI: 0.80 to 34.50) were numerically, but not statistically significantly related to sUA target achievement, and there was further no relationship between allopurinol or febuxostat doses with flare occurrence in year 2.

When different levels of target achievement at year 1 (<300 µmol/L vs 300 to <360 µmol/L vs >360 µmol/L) were compared with respect to flare occurrence at year 2, no major numeric or any statistically significant differences were seen.

## Discussion

In our patients with gout, who were educated on disease prevention and treated with ULT, BMI and the other anthropometric measures did not decline over 2 years. Cholesterol and LDL-C declined over the first year and remained mainly unchanged during year 2. Anthropometric measures at baseline were in general worse in patients with poor disease outcomes, tophus at baseline and not achieving the 2-year sUA target. Further, high HDL-C levels predicted a good outcome (sUA target <360 µmol/L) at year 2 and high LDL-C levels predicted a poor outcome with a flare during year 2. None of the classic lifestyle factors alcohol use, smoking, consumption of sugar-sweetened drinks or low physical activity independently predicted achievement of the sUA treatment target after 2 years or flares.

We had expected that focus on lifestyle and awareness for the disease would in patients manifest with a healthier lifestyle and decreased BMI. Changes in BMI over 2 years were only minimal and were with a slight increase of 0.4 units much lower than what could be considered as a minimal clinically important change.^29^ Possibly, the effective ULT strategy let patients experience reduced sUA levels during monthly visits and could thus have been considered sufficient by patients. Further, disease education by the nurse was primarily given during one consultation at baseline only and was not sufficient to promote longitudinal lifestyle changes among patients. On the other hand, we observed an improved lipid profile during year 1 with reduced cholesterol and LDL-C.

The observed association of lipid levels in our study with both longitudinal sUA achievement and reduced flare is novel but can only be considered exploratory. As high HDL-C is generally considered beneficial and high LDL-C poor, the predictive ability acted in the same direction, with high HDL-C predicting a good sUA outcome and high LDL-C predicting flare.

An increase of lipids during immunosuppressive treatment with biological disease-modifying antirheumatic drugs is known in inflammatory rheumatic disease,^30^ and decreased total cholesterol and HDL-C were observed in patients with active rheumatoid arthritis as compared with patients in remission.^31^ In our study, lipid levels decreased mainly during year 1 when inflammation, measured by ESR, also decreased. Elevated lipid levels were also seen in patients with tophi. We have earlier described increased levels of inflammation with increased calprotectin in our gout cohort^32^ with a relationship to large tophi as a poor disease outcome. Tight-control treat-to-target intervention was no longer performed after year 1 which could explain why changes of lipid levels especially primarily observed during year 1.

Alcohol consumption was recently linked to tophi^33^ and to a poor sUA outcome^22^ in gout, and had also observed by us after 1 -year,^24^ but alcohol consumption did not predict the 2-year sUA outcome. A familial interaction between obesity and alcohol consumption has been found to contribute to gout.^34^ Sugar-sweetened drinks reduced the chance of achieving the 2-year sUA target in multivariate analyses adjusting for some factors in our study but lost statistical significance in the final fully adjusted model together with other predictors.

Latourte *et al* found in a cross-sectional French study^22^ that low HDL-C levels and high total cholesterol, as well as higher WC (but not BMI), were associated with a poor ULT outcome, while in another study higher BMI predicted poor ULT response.^34^ In a Taiwanese cross-sectional study on individuals with hyperuricaemia or gout, total cholesterol and LDL-C were higher and HDL-C lower in poorly (sUA>6 mg/dL) versus well controlled individuals (sUA <6 mg/dL),^21^ and BMI/WC categories were independently related to sUA levels. In a mixed population with hyperuricaemia and gout, higher BMI was a predictor of poor response to ULT, but in contrast to our study lipid levels were only bivariately and not independently associated with ULT response.^35^

For flare occurrence, we lack established anthropometric and lipid predictors^36^; a small study in patients with acute stroke found an association between gout attacks and presence of hypercholesterolaemia.^37^ Thus, as far as we are aware, ours is the first longitudinal study to compare lifestyle factors, anthropometric measures and lipid levels with various gout outcomes. A strength is inclusion of a large number of actively treated patients with gout who were examined at multiple time points over 2 years, applying three different measures for disease severity.

Several limitations in our study deserve mentioning. The study was performed in a setting with tight-control ULT in one specialised rheumatology department with intensive ULT and an educational consultation with a nurse. This is a clinical situation which at present is not a care model for most patients with gout, and study findings need to be interpreted in that context. Further, we did not register use of statins in all our patients and use of statins may have contributed to low lipid levels. Also, we have no control population to compare our findings with. Finally, our study was observational which precludes causal conclusions.

In conclusion, BMI and other anthropometric measures were not improved over 2 years after intensive ULT and patient education. We found that modifiable factors—anthropomorphic and lipid variables—predicted gout outcomes with achieving both the sUA target and flares. Our findings give further support to recommended lifestyle changes in patients with gout, but more research is needed on the role of lipids in gout, and on the best provision of education to these patients.

## Data Availability

Data are available upon reasonable request.
